# Evaluation of the Effectiveness of a Cardiac Telerehabilitation Program in Chronic Heart Failure: Design and Rationale of the TELEREHAB-HF Study

**DOI:** 10.3390/healthcare13162074

**Published:** 2025-08-21

**Authors:** Marina Garofano, Carmine Vecchione, Mariaconsiglia Calabrese, Maria Rosaria Rusciano, Valeria Visco, Giovanni Granata, Albino Carrizzo, Gennaro Galasso, Placido Bramanti, Francesco Corallo, Lucia Pepe, Luana Budaci, Michele Ciccarelli, Alessia Bramanti

**Affiliations:** 1 Department of Medicine, Surgery and Dentistry, University of Salerno, 84084 Fisciano, Italy; cvecchione@unisa.it (C.V.); macalabrese@unisa.it (M.C.); mrusciano@unisa.it (M.R.R.); vvisco@unisa.it (V.V.); ggranata@unisa.it (G.G.); acarrizzo@unisa.it (A.C.); ggalasso@unisa.it (G.G.); mciccarelli@unisa.it (M.C.); 2 Faculty of Psychology, University eCampus, 22060 Novedrate, Italy; bramanti.dino@gmail.com; 3 Centro Neurolesi Bonino Pulejo, 98124 Messina, Italy; francesco.corallo@irccsme.it; 4 Rehabilitation Department, Azienda Ospedaliero-Universitaria “San Giovanni di Dio e Ruggi d’Aragona”, Via San Leonardo, 84125 Salerno, Italy; luciapepe88@gmail.com (L.P.); lu.budaci@gmail.com (L.B.)

**Keywords:** chronic heart failure, cardiac rehabilitation, tele-rehabilitation, exercise training, remote monitoring, home-based rehabilitation, digital health

## Abstract

**Background:** Despite strong guideline recommendations, participation in cardiac rehabilitation (CR) among patients with chronic heart failure (CHF) remains low due to logistical, geographical, and psychosocial barriers. Telerehabilitation may help overcome these limitations by offering remote, structured exercise programs supported by digital technologies. **Objective:** The TELEREHAB-HF study aims to evaluate the efficacy of an 8-week, home-based cardiac telerehabilitation program compared to standard in-person rehabilitation in patients with CHF. **Methods:** This is a prospective, controlled cohort study involving 220 adult patients with CHF (NYHA class I–III) clinically stable and on optimized therapy. Participants are assigned to either a telerehabilitation group (remote CR via a digital platform with wearable sensors and real-time physiotherapist supervision) or a standard in-person rehabilitation group. The primary outcome is the change in peak oxygen uptake (VO_2_max) at 8 weeks. Secondary outcomes include quality of life, functional performance, biochemical and echocardiographic parameters, and cognitive function, assessed at baseline and at 4, 8, 16, and 24 weeks. **Expected Results**: We hypothesize that telerehabilitation will be non-inferior to standard CR in improving functional capacity and secondary outcomes, with additional benefits in accessibility and adherence. Data from remote monitoring may also support a translational “rehabilomics” approach to exploring exercise-induced biomarker changes. **Conclusions:** This study seeks to assess the clinical effectiveness, safety, and feasibility of a home-based telerehabilitation model for CHF, with the goal of informing future strategies for broader implementation and personalized rehabilitation. Trial Registration: ClinicalTrials.gov Identifier: NCT07023536

## 1. Background

Chronic heart failure (CHF) remains a major public health concern due to its increasing prevalence, high rates of hospital readmissions, and significant impact on patient morbidity and healthcare costs. Cardiac rehabilitation (CR) is a cornerstone in the management of CHF, aiming to improve functional capacity, reduce symptoms, and enhance quality of life (QoL) [1,2]. Despite its well-established benefits and strong recommendations from international guidelines, the uptake of CR remains suboptimal, often due to logistical, geographical, or psychosocial barriers [3,4]. In recent years, digital health technologies, including telerehabilitation, a subset of telemedicine that leverages information and communication technologies (ICT) to deliver rehabilitation services remotely, have emerged as promising tools to overcome these limitations [5]. This approach offers the opportunity to extend access to structured exercise programs and clinical monitoring beyond the hospital setting, while maintaining the standards of care required for patients with complex chronic conditions. Preliminary studies and pilot programs suggest that cardiac telerehabilitation (CTR) may offer clinical outcomes comparable to those of conventional center-based rehabilitation, with added advantages in terms of flexibility, patient engagement, and healthcare resource optimization [6]. Recent developments in remote care delivery and digital rehabilitation frameworks have contributed to a growing interest in structured, home-based programs that integrate technology-driven monitoring and personalized training components [7]. Nevertheless, high-quality evidence from real-world settings is still needed to support widespread implementation and to better understand which patient populations may benefit most from remote interventions [8,9].

Despite the growing body of evidence supporting cardiac telerehabilitation (CTR), current implementations still present important limitations. Most existing models are based on asynchronous exercise prescriptions, relying on self-reported adherence or commercial-grade devices with limited clinical validation [6,10,11]. These programs typically monitor a narrow set of parameters (e.g., heart rate or steps) without real-time supervision, which limits their ability to adjust exercise intensity based on physiological responses or to ensure patient safety [7,11,12]. Moreover, outcome measures are often restricted to functional performance, with few studies incorporating longitudinal assessments of biochemical, echocardiographic, or cognitive parameters [7].

In addition, current CTR protocols rarely consider the molecular dimension of exercise-based rehabilitation. The potential role of physical activity in modulating biomarker profiles—such as natriuretic peptides or inflammatory markers—remains largely unexplored in remote settings, and very few programs include the collection of biospecimens to analyze individual biological responses to training.

To address these limitations, the TELEREHAB-HF study introduces a technologically advanced, multidimensional, and translational approach. Through the integration of the Khymeia VRRS [13,14,15,16]—a clinically validated platform that enables real-time video supervision and multidomain physiological monitoring—our protocol ensures both remote safety and exercise accuracy. Moreover, by incorporating a “rehabilomics” framework [17,18], the study will investigate the biological correlates of training, thereby supporting the development of personalized rehabilitation pathways.

In summary, the TELEREHAB-HF study aims not only to confirm the clinical efficacy of home-based cardiac rehabilitation, but also to expand the scientific understanding of its impact across physiological, functional, cognitive, and molecular domains—bridging the gap between digital innovation and translational cardiology.

This protocol describes a prospective, controlled study designed to evaluate the efficacy of an 8-week home-based cardiac telerehabilitation program, supported by real-time remote monitoring technologies, in patients with CHF. The primary objective is to assess improvements in exercise capacity, measured via peak oxygen uptake (VO_2_max), compared to standard in-hospital rehabilitation. Secondary outcomes will include biochemical, functional, and QoL measures.

In addition, the study adopts a translational and “rehabilomic” perspective, aiming to explore the role of exercise as a modulator of specific molecular pathways involved in heart failure [19,20]. By identifying circulating biomarkers that are both pathophysiologically relevant and potentially responsive to physical training, the study seeks to contribute to the development of personalized rehabilitation strategies and a deeper molecular characterization of CHF patients [21].

## 2. Methods

### Study Design and Population

**Figure 1:**
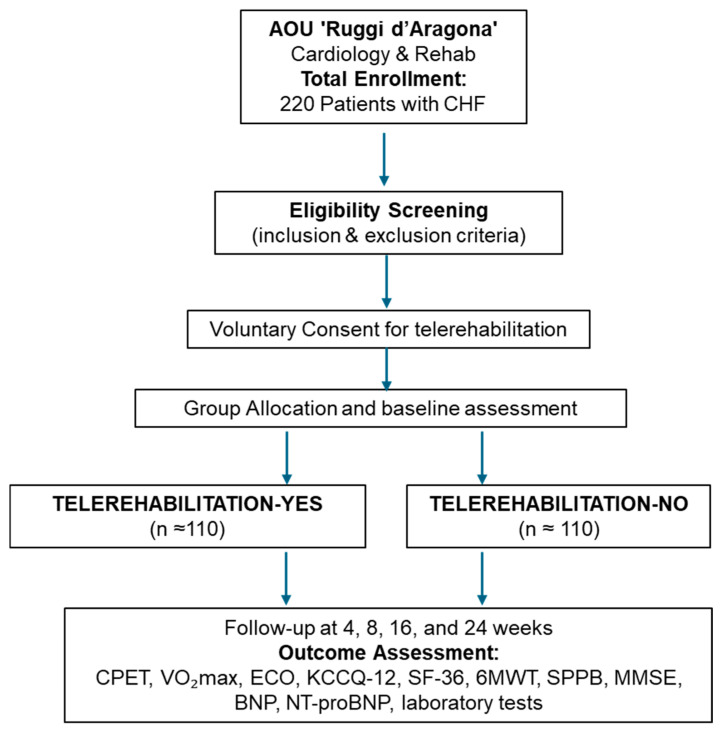
Flow diagram of the study design. Abbreviations: 6MWT: 6-Minute Walk Test; BNP: B-type Natriuretic Peptide; CPET: Cardiopulmonary Exercise Testing; ECHO: Echocardiography; KCCQ-12: Kansas City Cardiomyopathy Questionnaire—12 items; MMSE: Mini-Mental State Examination; NT-proBNP: N-terminal pro B-type Natriuretic Peptide; SF-36: Short Form Health Survey—36 items; SPPB: Short Physical Performance Battery; VO_2_max: Peak Oxygen Uptake.

The TELEREHAB-HF study is a single-center, prospective, observational, non-pharmacological cohort trial designed to evaluate the clinical efficacy of a home-based, supervised cardiac telerehabilitation program in patients with CHF. The trial will be conducted at the tertiary care center Azienda Ospedaliera Universitaria (AOU) “San Giovanni di Dio e Ruggi d’Aragona” in Salerno, Italy, in collaboration with the University of Salerno. The study aims to enroll 220 adult patients (≥18 years) with a confirmed diagnosis of CHF—classified as heart failure with reduced ejection fraction (HFrEF), heart failure with mildly reduced ejection fraction (HFmrEF), or heart failure with preserved ejection fraction (HFpEF)—and functional class I to III according to the New York Heart Association (NYHA) classification. All participants must be clinically stable and on optimized medical therapy for at least one month prior to enrollment (Figure 1).

Participants will be allocated to the telerehabilitation or standard rehabilitation arm based on patient preference and logistical eligibility (e.g., home safety, access to digital devices, internet connectivity). Although this approach does not involve randomization, both groups will be enrolled under identical inclusion and exclusion criteria. To mitigate potential selection bias, comprehensive baseline characterization and statistical adjustment (e.g., propensity score methods) will be applied. Patients in the intervention group (TELEREHABILITATION-YES) will undergo an 8-week individualized cardiac rehabilitation program performed at home, supported by the Khymeia Virtual Reality Rehabilitation System (VRRS). The program includes real-time video-supervised sessions with a physiotherapist, and continuous monitoring through medical-grade wearable sensors that collect electrocardiogram (ECG), heart rate (HR), peripheral oxygen saturation (SpO_2_), and blood pressure (BP) data. The system also records exercise performance using inertial movement sensors, and all data are transmitted securely to a central digital platform for remote clinical oversight and feedback.

The collected clinical and biometric data will contribute to a translational and “rehabilomics” [17,18] framework, aiming to explore the impact of exercise on the molecular profile of CHF patients. Blood and urine samples will be collected and stored in a biobank for future analysis of biomarkers potentially modifiable by physical activity, thus supporting the identification of individualized rehabilitation strategies.

Patients in the control group (TELEREHABILITATION-NO) will follow a conventional 8-week hospital-based cardiac rehabilitation program under direct supervision. Sessions will be conducted at the outpatient cardiac rehabilitation facility and will include the same structure and intensity of exercise training as the intervention group. Vital signs and effort perception will be assessed and recorded manually using standard clinical tools.

All patients, regardless of group assignment, will undergo standardized evaluations at baseline, and at 4, 8, 16, and 24 weeks.

Prior to the intervention, each participant will undergo a comprehensive clinical evaluation performed by a cardiologist specialized in cardiac rehabilitation—who also serves as the head of the Cardiac Rehabilitation Unit at AOU “San Giovanni di Dio e Ruggi d’Aragona” in Salerno—including clinical examination, ECG, transthoracic echocardiography, and cardiopulmonary exercise testing (CPET) [22,23].

In parallel, physiotherapists will conduct a set of standardized functional assessments, including: 6-Minute Walk Test (6MWT), Short Physical Performance Battery (SPPB), Mini-Mental State Examination (MMSE) for cognitive screening, administration of health-related quality of life (HRQoL) questionnaires, including the Kansas City Cardiomyopathy Questionnaire—12 items (KCCQ-12) and the Short Form Health Survey—36 items (SF-36) [24,25,26,27].

This multidisciplinary assessment ensures that all participants are clinically stable, functionally and cognitively evaluated, and safely eligible to participate in either arm of the rehabilitation program.

Subsequent assessments will include laboratory analyses, including B-type natriuretic peptide (BNP), N-terminal pro B-type natriuretic peptide (NT-proBNP), Serum creatinine, electrolytes (sodium, potassium, chloride), lipid profile, blood glucose.

Additionally, anthropometric measurements—body weight, height, and body mass index (BMI)—will be recorded at each timepoint to monitor weight dynamics, which are clinically relevant indicators of fluid retention and potential decompensation in patients with heart failure [2,28].

Patients with NYHA class IV symptoms, severe renal impairment—defined as an estimated glomerular filtration rate (eGFR) < 30 mL/min/1.73 m^2^—terminal illness, pregnancy, inability to perform physical training, or insufficient digital literacy (in the absence of caregiver support) will be excluded (Table 1).

All clinical, instrumental, and biochemical data will be recorded in anonymized case report forms and stored in a secure, encrypted digital database. The study protocol complies with the Declaration of Helsinki and Good Clinical Practice (GCP) guidelines and has been approved by the Campania 2 Regional Ethics Committee (Approval Number: 14718 of 06.06.2024). The trial is registered at ClinicalTrials.gov under the identifier NCT07023536.

## 3. Groups and Interventions

Participants enrolled in the study will be allocated to one of two groups based on their voluntary acceptance of the telerehabilitation modality:

1. TELEREHABILITATION—YES (Remote Group)
2. TELEREHABILITATION—NO (In-Person Group)

Both groups will undergo an 8-week individualized CR program based on international guidelines for heart failure rehabilitation [29]. The intervention includes a combined training protocol delivered 5 times per week for the first 4 weeks, and 3 times per week for the subsequent 4 weeks. Each session lasts approximately 60 min and is structured as follows (Table 2):

- 10 min of interval training (warm-up) including flexibility, breathing, and coordination exercises;
- 40 min of endurance training using a stationary bicycle, aiming at 70% of VO_2_ peak (as determined by baseline CPET) or 75–80% of maximum HR if CPET is not available [30,31,32,33,34];
- 10 min of cool-down exercises with stretching and breathing techniques.

## 4. In-Person Group (TELEREHABILITATION—NO)

Sessions are conducted under the direct supervision of a physiotherapist at the cardiac rehabilitation unit of the A.O.U. San Giovanni di Dio e Ruggi d’Aragona. Vital parameters (BP, HR, SpO_2_) are manually recorded; perception of fatigue and dyspnea is assessed using the Borg and Rate of Perceived Exertion (RPE) scales [23,35]. No technological devices are used for remote monitoring.

## 5. Remote Group (TELEREHABILITATION—YES)

Sessions are delivered synchronously at the patient’s home via a digital telerehabilitation platform (Khymeia VRRS Home System), enabling real-time audiovisual interaction with the physiotherapist. Patients are equipped with a home-based kit including a tablet, wearable sensors (K-RING, K-SENSOR), a spirometer (K-SPIRO), and a health monitor capable of recording ECG, HR, SpO_2_, and BP. The sensors adopted in the TELEREHAB-HF protocol were selected to ensure clinically relevant, real-time, and multidimensional monitoring of patients during home-based cardiac rehabilitation. These sensors were preferred over alternative solutions (e.g., consumer-grade smartwatches, standalone wearable ECG monitors, or optical motion capture systems) due to their medical-grade certification, full integration with the Khymeia VRRS platform, and ability to support both synchronous feedback and asynchronous data collection. Their interoperability, reliability, and user-friendliness in non-clinical environments make them particularly suitable for patients with chronic cardiovascular conditions undergoing home-based telerehabilitation.

All data are transmitted and stored securely in compliance with data protection regulations. Movement execution is monitored through inertial sensors, and real-time feedback is provided to ensure exercise accuracy. Borg and RPE scales are administered online during sessions.

In both groups, outcome measures (Anthropometric measures, CPET, 6MWT, SPPB, echocardiography, QoL questionnaires, laboratory tests, and MMSE) will be collected at baseline (T = 0), during the intervention (T = 1, at 4 weeks), post-intervention (T = 2, at 8 weeks), and during follow-up at 16 (T = 3) and 24 weeks (T = 4). Patients in the telerehabilitation group experiencing clinical or technical issues may transition to in-person sessions to ensure therapeutic continuity.

## 6. Technological Solution Used for the TELEREHABILITATION—YES Group

The technology used to deliver the telerehabilitation program is the Khymeia System (Virtual Reality Rehabilitation System, VRRS by Khymeia Group, Noventa Padovana, Italy; https://khymeia.com/it/ accessed on 24 February 2025). It is a Class I medical device designed for remote rehabilitation.

During the initial in-person training phase with the VRRS EVO system, the following components and accessories are used.

**Figure 2:**
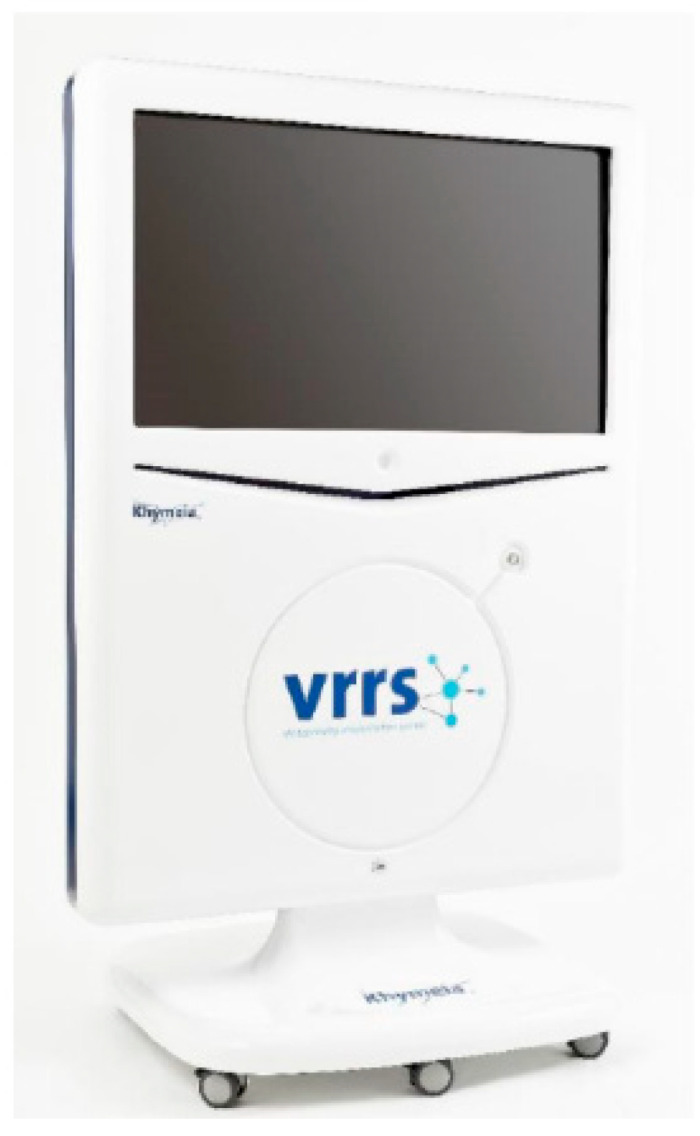
VRRS EVO.

VRRS EVO system (Figure 2), which includes:

- A processing unit enclosed in a dedicated cabinet, a capacitive touchscreen LCD monitor, and a low-intensity electromagnetic field generator
- Wireless 3D passive sensors worn by the patient, detecting position and orientation (6 degrees of freedom) through the electromagnetic field
- The number of sensors is adapted to specific rehabilitation needs

K-SENSOR: inertial sensors for monitoring movement parameters
K-Sensor bands: to support accurate and easy placement of sensors on the limbs during exercises
K-RING: a wearable sensor for continuous monitoring of HR and SpO_2_
K-SPIRO: a spirometer for guided respiratory exercises

Health Monitor: for measuring, displaying, and recording body temperature, BP, and single-lead ECG in home or clinical settings.

**Figure 3:**
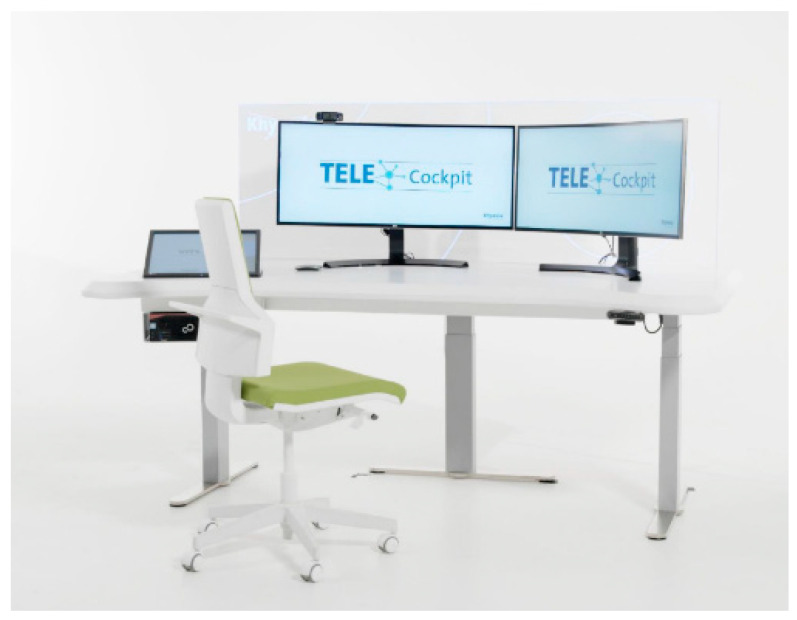
TeleCockpit System.

During the home-based physiotherapy program, following the in-person familiarization with VRRS EVO, the following components are also used:

TeleCockpit System (Figure 3): a high-tech control station for managing all telerehabilitation-related processes, protocols, and sessions. It includes: a height-adjustable, motorized workstation; a control terminal with keyboard and mouse for managing video conferencing and remote control sessions; dual-monitor setup; professional-grade audio-video conferencing system with camera and microphone; professional remote control system.

**Figure 4:**
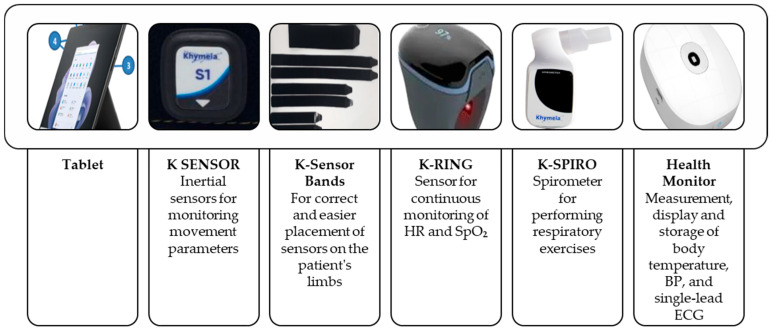
VRRS Home Kit. Abbreviations: BP: blood pressure, ECG: electrocardiogram, HR: heart rate, SpO_2_: peripheral capillary oxygen saturation.

VRRS Home Kit (Figure 4), enabling home telerehabilitation. This includes:

- Tablet device
- K-SENSOR inertial sensors
- K-Sensor bands for proper placement on limbs
- K-RING for continuous HR and SpO_2_
- K-SPIRO spirometer for breathing exercises
- Health Monitor for measuring and storing body temperature, BP, and ECG (single lead)

The Khymeia platform is also equipped with a Kloud service module, enabling full implementation of web-based telerehabilitation services with both synchronous and asynchronous functionality. Key features include:

- Continuous online updates of connected devices within the hub and spoke network
- Centralized sharing of clinical protocols and activities across all devices
- Real-time sharing and visualization of patient rehabilitation results
- Remote device allocation and control
- Assignment of personalized treatment programs and monitoring of adherence

The telerehabilitation system is modular and supports both clinical and home settings. It has built-in connectivity and enables secure cloud storage of all performed activities. To facilitate ease of use and maximize patient autonomy, all wearable sensors included in the VRRS Home Kit (e.g., K-RING, K-SENSOR) are designed for intuitive application. Each sensor is paired with pre-formed elastic bands or color-coded guides that ensure correct placement on the limbs or chest. During the initial in-person training session, physiotherapists provide step-by-step demonstrations and practical instructions on how to wear and activate the devices. Additionally, the VRRS offers on-screen visual aids and video tutorials accessible at any time during the home-based sessions. For participants with limited digital or motor skills, caregiver support is required, and real-time remote technical assistance is available to troubleshoot any difficulties. These features are intended to reduce the learning curve, improve adherence, and ensure accurate physiological data collection in a non-clinical environment.

## 7. Risk Management and Contingency Planning

To ensure patient safety and address potential risks associated with home-based cardiac telerehabilitation, a structured risk management strategy has been integrated into the TELEREHAB-HF protocol.

Each telerehabilitation session is supervised in real-time by a trained physiotherapist through a digital platform that continuously monitors vital signs using wearable medical-grade sensors. The system tracks SpO_2_, HR, and BP, and provides automatic alerts when pre-defined clinical thresholds are exceeded.

Safety thresholds triggering immediate session interruption and medical evaluation include [1,2,28]:

- Borg Dyspnea Scale ≥ 8/10
- Rate of Perceived Exertion ≥ 18/20
- Resting HR > 120 bpm or <50 bpm
- Systolic BP > 180 mmHg or <70 mmHg
- Diastolic BP > 100 mmHg or <50 mmHg
- SpO_2_ < 88%
- Sudden fall, acute chest pain, neurological symptoms (e.g., diplopia, motor/sensory deficits, aphasia), altered consciousness

In such events, the physiotherapist halts the session immediately and activates the emergency response protocol, which may include referral to the cardiologist or activation of local emergency medical services (EMS/118).

In case of technical malfunctions or persistent connectivity issues, a fallback mechanism is in place: patients are offered a transition to standard in-person rehabilitation sessions to ensure therapeutic continuity.

All patients receive initial in-person training to become familiar with the devices and the protocol. Those with limited digital literacy are required to have caregiver support as a condition for inclusion. Continuous technical assistance is available throughout the intervention period.

Clinical and biometric data are stored in a secure, encrypted repository, compliant with data protection regulations, ensuring traceability and safety monitoring.

**Figure 5:**
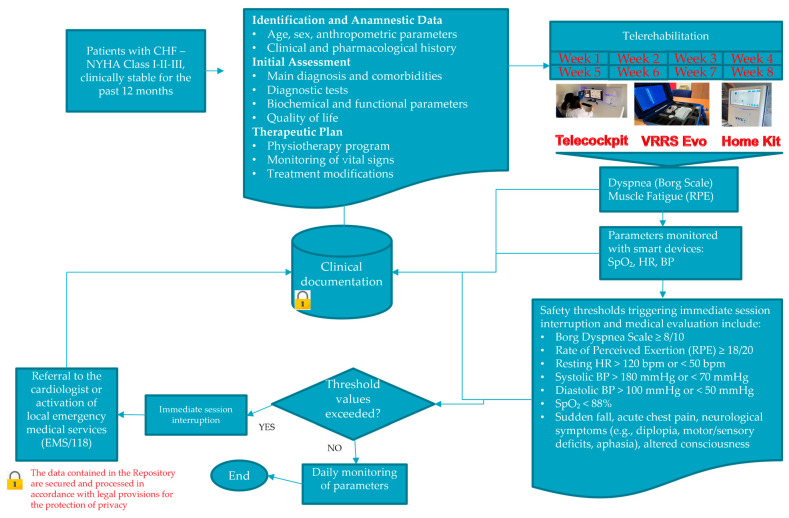
Emergency management flowchart within a tele-rehabilitation program for clinically stable CHF patients. Abbreviations: BNP: B-type Natriuretic Peptide, BP: Blood Pressure, CHF: Chronic Heart Failure, CPET: Cardiopulmonary Exercise Testing, EMS/118: Emergency Medical Services (Italian emergency number 118), HR: Heart Rate, KCCQ-12: Kansas City Cardiomyopathy Questionnaire (12 items), NYHA: New York Heart Association, RPE: Rating of Perceived Exertion, SF-36: Short Form Health Survey (36 items), SpO**_2_**: Peripheral Oxygen Saturation, VO**_2_**max: Maximal Oxygen Uptake, 6MWT: Six-Minute Walk Test.

This integrated safety protocol and contingency plan aim to minimize clinical and operational risks, optimize adherence, and ensure high standards of care throughout the rehabilitation pathway (Figure 5).

## 8. Outcomes

Both groups will undergo standardized assessments at baseline (T0), mid-treatment (4 weeks, T1), post-treatment (8 weeks, T2), and at follow-up timepoints (16 weeks, T3; 24 weeks, T4). Evaluations will include cardiopulmonary functional performance, echocardiographic measurements, biochemical markers, cognitive function, and QoL (Table 3).

The primary outcome of the study is the improvement in functional capacity, assessed as a ≥10% increase in VO_2_max, measured via CPET at 8 weeks (T2).

Secondary outcomes include:

- QoL: Assessed using the KCCQ-12 and SF-36 at each timepoint.
- Biochemical parameters: Changes in BNP, NT-proBNP, creatinine, eGFR, serum electrolytes (sodium, potassium, chloride), glucose, and lipid profile.
- Functional assessments: Performance on the 6MWT and SPPB.
- Cognitive status: Assessed with the MMSE.
- Echocardiographic parameters: Left ventricular ejection fraction (LVEF), diastolic function indices (E/A—Ratio of early E to late A ventricular filling velocities; E/e’—Ratio of early transmitral flow velocity to early diastolic mitral annular velocity), and right ventricular function (TAPSE—Tricuspid Annular Plane Systolic Excursion, RVs’—Right Ventricular Systolic Velocity).
- Adverse events: Monitoring of any complications related to training or telemonitoring (e.g., hypotension, arrhythmias, device issues).

These outcome measures will allow for a comprehensive and longitudinal evaluation of the effects of both in-person and telehealth cardiac rehabilitation on patients with chronic heart failure.

## 9. Statistical Analysis

### 9.1. Primary Endpoint Analysis

To evaluate the primary endpoint of the study—defined as a ≥10% improvement in VO_2_max at 8 weeks—paired comparisons between baseline and post-intervention values will be performed using a paired t-test for normally distributed data [36,37,38]. For non-normally distributed data, the Wilcoxon Signed-Rank test will be applied. Additionally, the proportion of patients achieving the predefined VO_2_max improvement threshold will be analyzed using logistic regression, with “Rehabilitation Type (Telerehabilitation vs. Standard)” as the main predictor. The model will be adjusted for clinically relevant covariates identified through baseline comparisons using ANOVA or chi-square tests. Odds ratios and 90% confidence intervals will be reported.

### 9.2. Secondary Endpoint Analysis

Continuous variables will be presented as mean ± standard deviation for normally distributed variables, and as median and interquartile range for those with non-normal distribution. Categorical data will be expressed as absolute numbers and percentages. The Shapiro–Wilk test will be used to assess normality.

- For repeated measurements (e.g., QoL scores, biochemical markers, functional parameters), comparisons across timepoints (T0, T1, T2, T3, T4) will be conducted using one-way repeated measures ANOVA for normally distributed data, or Friedman test for non-parametric data.
- For post hoc comparisons, Bonferroni correction will be applied to control for type I error.
- Categorical variables (e.g., proportion of patients with adverse events) will be compared using the Pearson chi-square test or Fisher’s exact test where appropriate.

A two-sided *p*-value < 0.05 will be considered statistically significant for all final analyses.

### 9.3. Sample Size Calculation

Based on the primary endpoint, the required sample size was calculated using G*Power software (Version 3.1.9.7). Assuming a 10% increase in VO_2_max in the intervention group compared to the control group, with a two-sided alpha level of 0.05 and statistical power (1–β) of 90%, the estimated sample size is 200 patients.

To compensate for a potential 10% dropout rate, the final target enrollment is 220 patients with CHF. This sample size is considered sufficient to detect clinically relevant differences in functional capacity and allows for robust evaluation of secondary outcomes including QoL, echocardiographic and biochemical parameters.

## 10. Expected Results

We expect to observe that the telerehabilitation group will demonstrate a significant improvement in functional capacity, as evidenced by a ≥10% increase in VO_2_max after 8 weeks of intervention, compared to the standard in-person rehabilitation group. This improvement is expected to be sustained at 24 weeks, indicating the long-term efficacy of the program. Furthermore, we expect to observe positive trends in secondary outcomes, including enhanced QoL (as measured by KCCQ-12 and SF-36), improved functional performance (6MWT, SPPB), and favorable changes in biochemical markers (BNP, NT-proBNP, creatinine, and electrolytes).

In the telerehabilitation group, the use of real-time monitoring and personalized feedback is also expected to improve adherence and safety, with a low incidence of adverse events. Additionally, we anticipate that echocardiographic parameters, such as LVEF and diastolic function indices (E/A, E/e’), will show measurable improvement or stabilization.

Overall, the study is expected to demonstrate that a structured, telerehabilitation program is not only clinically effective and safe, but also a feasible and scalable alternative to traditional CR for patients with CHF.

## 11. Discussion

The results of this study are expected to add to the existing evidence base regarding the use of telerehabilitation as a potential alternative to traditional center-based cardiac rehabilitation in patients with CHF. Observed improvements in functional capacity (VO_2_max), along with changes in QoL, functional performance, and selected biochemical and echocardiographic parameters, may help further clarify the role of exercise-based rehabilitation—whether delivered in-person or remotely—as part of comprehensive heart failure management.

Telerehabilitation presents certain advantages, such as increased accessibility, fewer logistical constraints, and the possibility of enhancing adherence through remote monitoring and real-time feedback [11,39]. These features may be particularly beneficial for individuals with limited mobility, transportation challenges, or those living in underserved or rural settings [40,41,42,43,44]. The use of wearable sensors and a secure digital platform allows for continuous monitoring and adjustment of exercise intensity, potentially supporting safety and protocol adherence. Should outcomes in the telerehabilitation group be comparable to, or better than, those of standard care, this could inform future considerations regarding its broader implementation and integration into healthcare systems seeking to optimize resource use and reduce hospital readmissions. A low rate of adverse events, coupled with satisfactory levels of patient engagement, may further support its feasibility.

Nonetheless, the study recognizes potential challenges, including variability in digital literacy, differences in home settings, and unequal access to technology [45,46]. To address these, participants will receive initial in-person training and continued technical assistance. Future investigations will be necessary to assess long-term clinical outcomes and cost-effectiveness beyond the six-month observation period [9].

In conclusion, this study seeks to evaluate the safety, feasibility, and potential utility of telerehabilitation as a component of secondary prevention and CHF management, in alignment with current clinical guidelines and digital health strategies.

## 12. Study Limitations and Future Directions

This study has several limitations that should be acknowledged. First, the single-center design may limit the generalizability of findings, as patients are recruited from a single university hospital with specific technological infrastructure and clinical pathways. Future multicenter trials are needed to validate the model in more diverse healthcare settings and populations.

Second, the study does not employ randomized allocation to intervention and control groups, which introduces the potential for selection bias. Although careful matching and baseline characterization are planned to mitigate this issue, a randomized controlled trial would offer a higher level of evidence and should be considered in future research phases. Nevertheless, this pragmatic allocation strategy is particularly relevant in the context of the Salerno province, which includes geographically complex and low-accessibility areas such as the Amalfi Coast and inland Cilento. These regions are often underserved in terms of rehabilitation services. By allowing patients to choose their rehabilitation modality, the protocol increases accessibility and adherence, reflecting the real-world feasibility of implementing home-based telerehabilitation programs in underserved or hard-to-reach populations.

Third, the duration of follow-up (24 weeks), while longer than many previous CTR studies, remains insufficient to evaluate long-term outcomes such as rehospitalizations, cardiovascular events, or mortality. Extended follow-up studies will be necessary to assess the sustained impact and safety of the intervention over time.

Fourth, although the study includes multidimensional clinical and biological assessments, it does not incorporate a formal cost-effectiveness analysis, which is crucial for informing health policy decisions and large-scale implementation. The economic sustainability of the proposed model should be evaluated in subsequent studies.

Fifth, while body weight, height, and BMI are routinely collected and monitored throughout the study, more advanced measures of body composition—such as visceral adiposity—are not included. Considering that fluctuations in body weight are a recognized predictor of heart failure decompensation, this parameter remains clinically relevant and will be carefully analyzed, although the lack of imaging-based adiposity assessment represents a limitation.

## Figures and Tables

**Table 1:** Inclusion and Exclusion Criteria.

Inclusion Criteria	Exclusion Criteria
Age ≥ 18 years	Age < 18 years
Confirmed diagnosis of CHF (HFrEF, HFmrEF, or HFpEF) NYHA functional class I–III	NYHA class IV
Clinically stable for ≥1 month On optimized medical therapy for ≥1 month	Terminal illness with life expectancy < 6 months Anticipated heart transplantation or VAD within 6 months Severe renal dysfunction (eGFR < 30 mL/min/1.73 m^2^)
Able to perform physical exercise	Inability to perform exercise due to medical conditions
Able to provide written informed consent	Severe cognitive impairment (e.g., advanced dementia)
Basic digital literacy (patient or caregiver, for tele-group)	No digital literacy and no caregiver (for tele-group)
Referred to cardiac rehabilitation	Participation in another clinical trial interfering with outcomes
—	Pregnancy or breastfeeding

Abbreviations: CHF: chronic heart failure, eGFR: estimated glomerular filtration rate, HFmrEF: heart failure with mildly reduced ejection fraction, HFpEF: heart failure with preserved ejection fraction, HFrEF: heart failure with reduced ejection fraction, NYHA: New York Heart Association, VAD: ventricular assist device.

**Table 2:** Comparison of Exercise Components Between Groups.

Exercise Component	TELEREHABILITATION—YES (Remote)	TELEREHABILITATION—NO (In-Person)
Warm-up (10 min)	- 3 min marching in place - 3 min cross-pattern exercises - 1 min sit-to-stand - 3 min breathing with Khymeia spirometer	- 3 min marching in place - 3 min cross-pattern exercises - 1 min sit-to-stand - 3 min breathing with volumetric incentive spirometer
Endurance Training (40 min)	stationary cycling	stationary cycling
Cool-down (10 min)	- 5 min stretching scapular/pelvic muscles - 5 min breathing with Khymeia spirometer	- 5 min stretching scapular/pelvic muscles - 5 min breathing with volumetric incentive spirometer

**Table 3:** Outcome Measures by Timepoint.

Outcome Type	Outcome Measure	Unit of Measure	Domain	Clinical Relevance/Rationale	T0 (Baseline)	T1 (4 wks)	T2 (8 wks)	T3 (12 wks)	T4 (24 wks)
Primary	≥10% improvement in VO_2_max (CPET)	mL/kg/min	Functional Capacity	Gold standard for evaluating aerobic capacity and predicting prognosis in CHF	✔		✔	✔	✔
Secondary	Change in body weight	Kg	Anthropometric	Important for monitoring nutritional status; weight fluctuations are clinically relevant predictors of CHF decompensation.	✔	✔	✔	✔	✔
Secondary	Change in BMI	kg/m^2^	Anthropometric	Static measure to stratify overweight, obesity, which may influence exercise response and prognosis in CHF	✔	✔	✔	✔	✔
Secondary	Change in KCCQ-12 Score	Points (0–100)	QoL	CHF-specific QoL instrument sensitive to clinical changes	✔	✔	✔	✔	✔
Secondary	Change in SF-36 Score	Points (0–100)	QoL	Generic QoL questionnaire for broader health-related QoL	✔	✔	✔	✔	✔
Secondary	Change in 6MWT	Meters	Functional	Test of submaximal exercise capacity; reflects improvements in daily functional status	✔	✔	✔	✔	✔
Secondary	Change in SPPB Score	Points (0–12)	Functional	Assesses lower extremity strength, balance, and mobility—predictive of disability and frailty	✔	✔	✔	✔	✔
Secondary	Change in MMSE	points (0–30)	Cognitive	Screens for cognitive impairment, which can affect adherence and prognosis in CHF patients.	✔	✔	✔	✔	✔
Secondary	Change in BNP	pg/mL	Biochemical	Established biomarkers of myocardial stress; reflect CHF severity and response to therapy.	✔		✔	✔	✔
Secondary	Change in NT-proBNP	pg/mL	Biochemical	Established biomarkers of myocardial stress; reflect CHF severity and response to therapy.	✔		✔	✔	✔
Secondary	Change in Serum Creatinine	mg/dL	Biochemical	Monitor renal function, critical in CHF management due to cardiorenal interactions and therapy impact	✔		✔	✔	✔
Secondary	Change in eGFR	mL/min/1.73 m^2^	Biochemical	Monitor renal function, critical in CHF management due to cardiorenal interactions and therapy impact	✔		✔	✔	✔
Secondary	Change in Serum Sodium	mmol/L	Biochemical	Important for assessing treatment safety (e.g., diuretics, ACE inhibitors) and arrhythmic risk	✔		✔	✔	✔
Secondary	Change in Serum Potassium	mmol/L	Biochemical	Important for assessing treatment safety (e.g., diuretics, ACE inhibitors) and arrhythmic risk	✔		✔	✔	✔
Secondary	Change in Serum Chloride	mmol/L	Biochemical	Important for assessing treatment safety (e.g., diuretics, ACE inhibitors) and arrhythmic risk	✔		✔	✔	✔
Secondary	Change in Glucose	mg/dL	Biochemical	Cardiovascular risk profile monitoring and potential metabolic benefits from exercise	✔		✔	✔	✔
Secondary	Change in Lipids	mg/dL	Biochemical	Cardiovascular risk profile monitoring and potential metabolic benefits from exercise	✔		✔	✔	✔
Secondary	Change in LVEF	%	Echocardiographic	Quantifies systolic and diastolic function; allows tracking of cardiac remodeling and hemodynamic response to rehabilitation	✔		✔	✔	✔
Secondary	Change in E/A and E/e’	Ratio	Echocardiographic	Quantifies systolic and diastolic function; allows tracking of cardiac remodeling and hemodynamic response to rehabilitation	✔		✔	✔	✔
Secondary	Change in TAPSE, RVs’	mm/cm/s	Echocardiographic	Quantifies systolic and diastolic function; allows tracking of cardiac remodeling and hemodynamic response to rehabilitation	✔		✔	✔	✔
Secondary	Adverse Events	Number of events over the total number of treatments administered	Safety	Monitors safety and tolerability of both telerehabilitation and standard rehabilitation interventions		✔	✔	✔	✔

Abbreviations: 6MWT—6-Minute Walk Test; ACE- Angiotensin-Converting Enzyme; BNP—B-type Natriuretic Peptide; CPET—Cardiopulmonary Exercise Testing; CHF—Chronic heart failure; E/A—Ratio of early (E) to late (A) ventricular filling velocities; E/e’—Ratio of early transmitral flow velocity to early diastolic mitral annular velocity; ECG—Electrocardiogram; eGFR—Estimated Glomerular Filtration Rate; KCCQ-12—Kansas City Cardiomyopathy Questionnaire—12 items; LVEF—Left Ventricular Ejection Fraction; MMSE—Mini-Mental State Examination; NT-proBNP—N-terminal pro B-type Natriuretic Peptide; QoL—Quality of life; SF-36—Short Form Health Survey—36 items; SPPB—Short Physical Performance Battery; TAPSE—Tricuspid Annular Plane Systolic Excursion; VO_2_max—Maximal Oxygen Uptake; RVs’—Right Ventricular Systolic Velocity; ✔—timepoints at which the outcome measure was assessed.

## Data Availability

The datasets generated and/or analyzed during the current study are not publicly available at this stage due to ongoing data collection but will be made available from the corresponding author upon reasonable request after study completion.

## References

[1] McDonagh T.A., Metra M., Adamo M., Gardner R.S., Baumbach A., Böhm M., Burri H., Butler J., Čelutkienė J., Chioncel O. (2021). 2021 ESC Guidelines for the diagnosis and treatment of acute and chronic heart failure. Eur. Heart J..

[2] Heidenreich P.A., Bozkurt B., Aguilar D., Allen L.A., Byun J.J., Colvin M.M., Deswal A., Drazner M.H., Dunlay S.M., Evers L.R. (2022). 2022 AHA/ACC/HFSA Guideline for the Management of Heart Failure: A Report of the American College of Cardiology/American Heart Association Joint Committee on Clinical Practice Guidelines. Circulation.

[3] Keteyian S.J., Jackson S.L., Chang A., Brawner C.A., Wall H.K., Forman D.E., Sukul D., Ritchey M.D., Sperling L.S. (2022). Tracking Cardiac Rehabilitation Utilization in Medicare Beneficiaries: 2017 UPDATE. J. Cardiopulm. Rehabil. Prev..

[4] Taylor R.S., Dalal H.M., Zwisler A.D. (2023). Cardiac rehabilitation for heart failure: ‘Cinderella’ or evidence-based pillar of care?. Eur. Heart J..

[5] Jansen-Kosterink S., In ‘t Veld R.H., Hermens H., Vollenbroek-Hutten M. (2015). A Telemedicine Service as Partial Replacement of Face-to-Face Physical Rehabilitation: The Relevance of Use. Telemed. J. E-Health.

[6] Frederix I., Solmi F., Piepoli M.F., Dendale P. (2017). Cardiac telerehabilitation: A novel cost-efficient care delivery strategy that can induce long-term health benefits. Eur. J. Prev. Cardiol..

[7] Garofano M., Vecchione C., Calabrese M., Rusciano M.R., Visco V., Granata G., Carrizzo A., Galasso G., Bramanti P., Corallo F. (2024). Technological Developments, Exercise Training Programs, and Clinical Outcomes in Cardiac Telerehabilitation in the Last Ten Years: A Systematic Review. Healthcare.

[8] Seron P., Oliveros M.J., Gutierrez-Arias R., Fuentes-Aspe R., Torres-Castro R.C., Merino-Osorio C., Nahuelhual P., Inostroza J., Jalil Y., Solano R. (2021). Effectiveness of Telerehabilitation in Physical Therapy: A Rapid Overview. Phys. Ther..

[9] Zhong W., Liu R., Cheng H., Xu L., Wang L., He C., Wei Q. (2023). Longer-Term Effects of Cardiac Telerehabilitation on Patients With Coronary Artery Disease: Systematic Review and Meta-Analysis. JMIR Mhealth Uhealth.

[10] Kraal J.J., Van den Akker-Van Marle M.E., Abu-Hanna A., Stut W., Peek N., Kemps H.M. (2017). Clinical and cost-effectiveness of home-based cardiac rehabilitation compared to conventional, centre-based cardiac rehabilitation: Results of the FIT@Home study. Eur. J. Prev. Cardiol..

[11] Scherrenberg M., Falter M., Abreu A., Aktaa S., Busnatu S., Casado-Arroyo R., Dendale P., Dilaveris P., Locati E.T., Marques-Sule E. (2025). Standards for cardiac telerehabilitation: A scientific statement of the European Association of Preventive Cardiology (EAPC) and the Association of Cardiovascular Nursing & Allied Professions (ACNAP) of the ESC, and the ESC Working Group on e-Cardiology. Eur. Heart J..

[12] Fang J., Huang B., Xu D., Li J., Au W.W. (2019). Innovative Application of a Home-Based and Remote Sensing Cardiac Rehabilitation Protocol in Chinese Patients After Percutaneous Coronary Intervention. Telemed. J. E-Health.

[13] Alemanno F., Houdayer E., Emedoli D., Locatelli M., Mortini P., Mandelli C., Raggi A., Iannaccone S. (2019). Efficacy of virtual reality to reduce chronic low back pain: Proof-of-concept of a non-pharmacological approach on pain, quality of life, neuropsychological and functional outcome. PLoS ONE.

[14] Macchitella L., Amendola S., Barraco G., Scoditti S., Gallo I., Oliva M.C., Trabacca A. (2023). A narrative review of the use of a cutting-edge virtual reality rehabilitation technology in neurological and neuropsychological rehabilitation. NeuroRehabilitation.

[15] Contrada M., Arcuri F., Tonin P., Pignolo L., Mazza T., Nudo G., Pignataro M.L., Quintieri M., Iozzi A., Cerasa A. (2021). Stroke Telerehabilitation in Calabria: A Health Technology Assessment. Front. Neurol..

[16] Galasso O., Calabrese M., Scanniello G., Garofano M., Pepe L., Budaci L., Ungaro G., Fimiani G., Bramanti P., Schiavo L. (2025). Accelerating Recovery: A Case Report on Telerehabilitation for a Triathlete’s Post-Meniscus Surgery Comeback. Healthcare.

[17] Wagner A.K. (2011). Rehabilomics: A conceptual framework to drive biologics research. PM R J. Inj. Funct. Rehabil..

[18] Wagner A.K. (2014). A Rehabilomics framework for personalized and translational rehabilitation research and care for individuals with disabilities: Perspectives and considerations for spinal cord injury. J. Spinal Cord. Med..

[19] Badianyama M., Mpanya D., Adamu U., Sigauke F., Nel S., Tsabedze N. (2022). New Biomarkers and Their Potential Role in Heart Failure Treatment Optimisation-An African Perspective. J. Cardiovasc. Dev. Dis..

[20] Mariappan V., Srinivasan R., Pratheesh R., Jujjuvarapu M.R., Pillai A.B. (2024). Predictive biomarkers for the early detection and management of heart failure. Heart Fail. Rev..

[21] Malandish A., Ghadamyari N., Karimi A., Naderi M. (2022). The role of exercise training on cardiovascular peptides in patients with heart failure: A systematic review and meta-analysis. Curr. Res. Physiol..

[22] Verdicchio C., Freene N., Hollings M., Maiorana A., Briffa T., Gallagher R., Hendriks J.M., Abell B., Brown A., Colquhoun D. (2023). A Clinical Guide for Assessment and Prescription of Exercise and Physical Activity in Cardiac Rehabilitation. A CSANZ Position Statement. Heart Lung Circ..

[23] Brown T.M., Pack Q.R., Aberegg E., Brewer L.C., Ford Y.R., Forman D.E., Gathright E.C., Khadanga S., Ozemek C., Thomas R.J. (2024). Core Components of Cardiac Rehabilitation Programs: 2024 Update: A Scientific Statement From the American Heart Association and the American Association of Cardiovascular and Pulmonary Rehabilitation. Circulation.

[24] Giannitsi S., Bougiakli M., Bechlioulis A., Kotsia A., Michalis L.K., Naka K.K. (2019). 6-minute walking test: A useful tool in the management of heart failure patients. Ther. Adv. Cardiovasc. Dis..

[25] Yamamoto S., Yamaga T., Nishie K., Sakai Y., Ishida T., Oka K., Ikegami S., Horiuchi H. (2020). Impact of physical performance on prognosis among patients with heart failure: Systematic review and meta-analysis. J. Cardiol..

[26] Brown K. (2003). A review to examine the use of SF-36 in cardiac rehabilitation. Br. J. Nurs..

[27] Sukosd I.E., Pescariu S.A., Faur C., Danila A.I., Prodan-Barbulescu C., Fira-Mladinescu O. (2024). Utility of Kansas City Cardiomyopathy Questionnaire (KCCQ) in Assessing Quality of Life among Patients with Heart Failure Undergoing Exercise Training Rehabilitation: A Systematic Review. Diseases.

[28] Virani S.S., Newby L.K., Arnold S.V., Bittner V., Brewer L.C., Demeter S.H., Dixon D.L., Fearon W.F., Hess B., Johnson H.M. (2023). 2023 AHA/ACC/ACCP/ASPC/NLA/PCNA Guideline for the Management of Patients With Chronic Coronary Disease: A Report of the American Heart Association/American College of Cardiology Joint Committee on Clinical Practice Guidelines. Circulation.

[29] Beatty A.L., Beckie T.M., Dodson J., Goldstein C.M., Hughes J.W., Kraus W.E., Martin S.S., Olson T.P., Pack Q.R., Stolp H. (2023). A New Era in Cardiac Rehabilitation Delivery: Research Gaps, Questions, Strategies, and Priorities. Circulation.

[30] Pelliccia A., Sharma S., Gati S., Bäck M., Börjesson M., Caselli S., Collet J.P., Corrado D., Drezner J.A., Halle M. (2021). 2020 ESC Guidelines on sports cardiology and exercise in patients with cardiovascular disease. Eur. Heart J..

[31] Piepoli M.F., Conraads V., Corrà U., Dickstein K., Francis D.P., Jaarsma T., McMurray J., Pieske B., Piotrowicz E., Schmid J.P. (2011). Exercise training in heart failure: From theory to practice. A consensus document of the Heart Failure Association and the European Association for Cardiovascular Prevention and Rehabilitation. Eur. J. Heart Fail..

[32] Corrà U., Piepoli M.F., Carré F., Heuschmann P., Hoffmann U., Verschuren M., Halcox J., Giannuzzi P., Saner H., Wood D. (2010). Secondary prevention through cardiac rehabilitation: Physical activity counselling and exercise training: Key components of the position paper from the Cardiac Rehabilitation Section of the European Association of Cardiovascular Prevention and Rehabilitation. Eur. Heart J..

[33] Sachdev V., Sharma K., Keteyian S.J., Alcain C.F., Desvigne-Nickens P., Fleg J.L., Florea V.G., Franklin B.A., Guglin M., Halle M. (2023). Supervised exercise training for chronic heart failure with preserved ejection fraction: A scientific statement from the American Heart Association and American College of Cardiology. Circulation.

[34] Gayda M., Ribeiro P.A.B., Juneau M., Nigam A. (2016). Comparison of Different Forms of Exercise Training in Patients With Cardiac Disease: Where Does High-Intensity Interval Training Fit?. Can. J. Cardiol..

[35] Borg G.A. (1982). Psychophysical bases of perceived exertion. Med. Sci. Sports Exerc..

[36] McGregor G., Powell R., Begg B., Birkett S.T., Nichols S., Ennis S., McGuire S., Prosser J., Fiassam O., Hee S.W. (2023). High-intensity interval training in cardiac rehabilitation: A multi-centre randomized controlled trial. Eur. J. Prev. Cardiol..

[37] Keteyian S.J., Brawner C.A., Savage P.D., Ehrman J.K., Schairer J., Divine G., Aldred H., Ophaug K., Ades P.A. (2008). Peak aerobic capacity predicts prognosis in patients with coronary heart disease. Am. Heart J..

[38] Baccanelli G., Tomaselli M., Ferri U., Giglio A., Munforti C., Parati G., Facchini M., Crotti L., Malfatto G. (2023). Effects of cardiac rehabilitation on cardiopulmonary test parameters in heart failure: A real world experience. Int. J. Cardiol. Cardiovasc. Risk Prev..

[39] Scherrenberg M., Zeymer U., Schneider S., Van der Velde A.E., Wilhelm M., Van’t Hof A.W.J., Kolkman E., Prins L.F., Prescott E., Iliou M.C. (2021). EU-CaRE study: Could exercise-based cardiac telerehabilitation also be cost-effective in elderly?. Int. J. Cardiol..

[40] Albarqi M.N. (2024). Exploring the Effectiveness of Technology-Assisted Interventions for Promoting Independence in Elderly Patients: A Systematic Review. Healthcare.

[41] Areias A.C., Molinos M., Moulder R.G., Janela D., Scheer J.K., Bento V., Yanamadala V., Cohen S.P., Correia F.D., Costa F. (2023). The potential of a multimodal digital care program in addressing healthcare inequities in musculoskeletal pain management. NPJ Digit. Med..

[42] Brouwers R.W.M., van Exel H.J., van Hal J.M.C., Jorstad H.T., de Kluiver E.P., Kraaijenhagen R.A., Kuijpers P., van der Linde M.R., Spee R.F., Sunamura M. (2020). Cardiac telerehabilitation as an alternative to centre-based cardiac rehabilitation. Neth. Heart J..

[43] Ezeamii V.C., Okobi O.E., Wambai-Sani H., Perera G.S., Zaynieva S., Okonkwo C.C., Ohaiba M.M., William-Enemali P.C., Obodo O.R., Obiefuna N.G. (2024). Revolutionizing Healthcare: How Telemedicine Is Improving Patient Outcomes and Expanding Access to Care. Cureus.

[44] Jack K., McLean S.M., Moffett J.K., Gardiner E. (2010). Barriers to treatment adherence in physiotherapy outpatient clinics: A systematic review. Man. Ther..

[45] Rawstorn J.C., Gant N., Rolleston A., Whittaker R., Stewart R., Benatar J., Warren I., Meads A., Jiang Y., Maddison R. (2018). End Users Want Alternative Intervention Delivery Models: Usability and Acceptability of the REMOTE-CR Exercise-Based Cardiac Telerehabilitation Program. Arch. Phys. Med. Rehabil..

[46] Cooper L.B., Mentz R.J., Sun J.L., Schulte P.J., Fleg J.L., Cooper L.S., Piña I.L., Leifer E.S., Kraus W.E., Whellan D.J. (2015). Psychosocial Factors, Exercise Adherence, and Outcomes in Heart Failure Patients: Insights From Heart Failure: A Controlled Trial Investigating Outcomes of Exercise Training (HF-ACTION). Circ. Heart Fail..

